# Using the *HER2/CEP17* FISH Ratio to Predict Pathologic Complete Response Following Neoadjuvant Anti-HER2 Doublet Therapy in HER2+ Breast Cancer

**DOI:** 10.1093/oncolo/oyac247

**Published:** 2022-12-10

**Authors:** Eric M Lander, Katherine C Rappazzo, Li-Ching Huang, Jiun-Ruey Hu, Heidi Chen, Yu Shyr, Vandana G Abramson

**Affiliations:** Department of Medicine, Division of Hematology/Oncology, Vanderbilt-Ingram Cancer Center, Vanderbilt University, Nashville, TN, USA; Department of Hematology/Oncology, Sidney Kimmel Comprehensive Cancer Center, Johns Hopkins University, Baltimore, MD, USA; Department of Biostatistics, Vanderbilt University Medical Center, Vanderbilt University, Nashville, TN, USA; Department of Internal Medicine, Yale School of Medicine, New Haven, CT, USA; Department of Biostatistics, Vanderbilt University Medical Center, Vanderbilt University, Nashville, TN, USA; Department of Biostatistics, Vanderbilt University Medical Center, Vanderbilt University, Nashville, TN, USA; Department of Medicine, Division of Hematology/Oncology, Vanderbilt-Ingram Cancer Center, Vanderbilt University, Nashville, TN, USA

**Keywords:** breast cancer, HER2, *HER2/CEP17* FISH ratio, pathologic complete response, trastuzumab, pertuzumab

## Abstract

**Background:**

Clinical trials of HER2-directed therapy that omit neoadjuvant conventional chemotherapy for HER+ breast cancer demonstrate that a subset of patients still obtains a pCR. Identifying tumor characteristics which predict pCR may help select patients for de-escalated neoadjuvant dual HER2-targeted treatment without chemotherapy. This is the first study evaluating the *HER2/CEP17* ratio by FISH as a biomarker to predict pCR among patients who received neoadjuvant anti-HER2 regimens without chemotherapy.

**Patients and Methods:**

Data from patients with locally advanced HER2+ breast cancer who received neoadjuvant dual HER2-targeted therapy without conventional chemotherapy from a single center was retrospectively reviewed. All patients were enrolled in one of 3 clinical trials evaluating chemotherapy de-escalation. Logistic regression modeling assessed for a relationship between the *HER2/CEP17* FISH ratio obtained from baseline tissue biopsy and pCR based on pathology at the time of definitive breast surgery following neoadjuvant treatment.

**Results:**

Following neoadjuvant treatment with dual HER2-targeted therapies in 56 patients, the probability of pCR was 73% among patients with a *HER2* ratio of 13.1 compared to a probability of 38% among patients with *HER2* ratio of 5.5 (OR 4.14, 95% CI 1.44-11.89; *P* = .012). This positive association persisted after controlling for different treatment regimens administered (OR 2.87, 95% CI 0.9-9.18, *P* = .020).

**Conclusions:**

These data found a positive association between the *HER2/CEP17* FISH ratio and pCR following neoadjuvant dual HER2-targeted therapy without chemotherapy. Larger prospective studies are needed to validate the *HER2* ratio as a biomarker to select patients for neoadjuvant dual anti-HER2 therapy without chemotherapy.

**Implications for Practice**

The *HER2/CEP17* FISH ratio is known to predict pCR for patients who receive neoadjuvant HER2-targeted therapy with chemotherapy, and it may help select patients with locally advanced HER2+ breast cancer for de-escalated neoadjuvant treatment that omits cytotoxic chemotherapy. These data found that patients with a higher *HER2/CEP17* FISH ratio from baseline tissue biopsy were more likely to achieve a pCR following neoadjuvant dual HER2-targeted therapy without chemotherapy.

## Introduction

Human epidermal growth factor receptor 2 (HER2) is a transmembrane receptor tyrosine kinase that is amplified in 18%-20% of human breast carcinomas and has been identified as a therapeutic target.^1-3^ The definition of HER2-positive (HER2+) breast cancer derives from quantification of the HER2 protein via immunohistochemistry (IHC) or fluorescence in situ hybridization (FISH) analyses of biopsy samples. IHC stains the HER2 protein on the cell surface and is graded from 0 to 3+, where HER2+ is defined as 3+ by IHC staining according to current ASCO/CAP guidelines.^4^ Alternatively, the *HER2/CEP17* ratio by FISH quantifies the number of *HER2* gene copies on chromosome 17 (*ERBB2*) in relation to the number of chromosome 17 centromere (*CEP17*) copies per nucleus, where HER2+ is a *HER2/CEP17* FISH ratio ≥ 2 or absolute *HER2* copy number ≥ 6.^4,5^ The *HER2/CEP17* FISH ratio has also been reported as the *HER2* ratio.^6^

Pathologic complete response (pCR), defined as no residual invasive tumor in the breast and axillary lymph nodes following neoadjuvant treatment, is associated with improved event-free and overall survival.^7-11^ The development of the HER2-targeted monoclonal antibody, trastuzumab (T), has led to considerably increased pCR rates and improved long-term outcomes of patients with HER2+ breast cancer.

Trastuzumab, in combination with chemotherapy, achieves pCR rates of 29%-40%.^9,12,13^ In 2013, pertuzumab, another monoclonal antibody targeting HER2, received approval for the treatment of locally advanced HER2+ breast cancer in combination with trastuzumab and chemotherapy. The approval was based on an increase in pCR rate from 29% with chemotherapy and trastuzumab to 46% with the addition of pertuzumab.^13^

Newer HER2-targeting drugs have also been approved for the treatment of HER2+ breast cancer. The tyrosine kinase inhibitors, lapatinib and neratinib, are approved for metastatic breast cancer.^14,15^ Several antibody-drug conjugates are also approved for metastatic HER2+ breast cancer. These include ado-trastuzumab emtansine, an antibody-drug conjugate which links trastuzumab to a microtubule inhibitor, fam-trastuzumab deruxtecan, a HER2 antibody conjugated to a topoisomerase inhibitor, and margituximab, a novel Fc-engineered anti-HER2 monoclonal antibody.^16,17^

The optimal neoadjuvant treatment strategy for HER2+ breast cancer is evolving. While HER2-targeting antibodies administered with cytotoxic chemotherapy offer the highest rates of pCR in the neoadjuvant setting, the regimens confer substantial side effects, financial toxicity, and potential over-treatment for a significant proportion of patients. Pre-clinical studies showed that a pCR can be achieved following dual anti-HER2 therapies without chemotherapy. As a result, de-escalation strategies to omit cytotoxic chemotherapy from neoadjuvant regimens have been investigated in the NeoSphere, TBCRC006, TBCRC023, and PAMELA trials.^13,18-20^ Ultimately, these trials of dual HER2-targeted therapy with varying treatment durations and varying combinations of anti-HER2 agents have shown the possibility of achieving pCR without chemotherapy (20%-40% of patients), but with fewer grade ≥ 3 toxicities.^21^ The selection of patients who may benefit from this de-escalation strategy versus additional chemotherapy remains unclear.

The proper identification of candidates for dual HER2-targeted therapy without chemotherapy remains a challenge and disease biomarkers may elucidate the subset of HER2+ breast cancers that are primarily dependent on HER2 signaling for growth and that may respond to HER2 directed therapies without chemotherapy. The *HER2* ratio may be an accessible method of identifying potential candidates for dual HER2-targeted therapy without chemotherapy, as tumors with increased *HER2* expression exhibit more oncogene reliance and subsequent improved response to HER2-targeted agents.^18,22,23^ Trials with neoadjuvant trastuzumab with chemotherapy have shown that a higher *HER2* ratio predicts pCR and recurrence-free survival.^24-26^ In contrast, one study of trastuzumab with chemotherapy noted impaired disease-free survival at the highest range of *HER2* ratio > 8.^27^ Veeraraghavan et al found that no patients with a *HER2* ratio 2-4 who had received anti-HER2 doublet therapy without chemotherapy achieved pCR, but 29% of patients with *HER2* ratio ≥ 4 achieved pCR.^6^

This novel exploratory study is the first to examine whether pathologic complete response following various neoadjuvant anti-HER2 doublet therapies without conventional chemotherapy is dependent on the level of *HER2* amplification. Based on prior clinical data in patients receiving trastuzumab with chemotherapy, we hypothesized that higher *HER2/CEP17* FISH ratios would be associated with a higher pCR rate in patients receiving dual anti-HER2 therapy without chemotherapy.^24-26^

## Materials and Methods

### Study Design and Sample

This study was a single-center retrospective cohort study. Patients at the Vanderbilt-Ingram Cancer Center who received neoadjuvant treatment for HER2-positive breast cancer were identified using the Vanderbilt University Medical Center (VUMC) Tumor Registry in Nashville, Tennessee. All 56 patients participated in one of 3 clinical trials evaluating HER2-directed therapy without chemotherapy at the Vanderbilt-Ingram Cancer Center between May 2009 and April 2017. Patient information from the database was verified via cross-referencing of patient electronic medical record data and was collected and managed using REDCap electronic data capture tools hosted at VUMC.^28^

Patients were included if they had histologically confirmed stages II-III invasive HER2+ breast cancer, received neoadjuvant dual HER2-targeted therapy without conventional chemotherapy, and underwent definitive breast surgery. No patients who met these criteria between the specified dates of the study at VUMC were excluded.

Anti-HER2 doublet treatment was given according to study protocols that included trastuzumab emtansine plus pertuzumab (T-DM1 + P) every 3 weeks for 18 weeks (6 cycles), trastuzumab plus pertuzumab (T + P) every 3 weeks for 12 weeks (4 cycles), and weekly trastuzumab plus daily lapatinib (T + L) for 12 or 24 weeks.^20,29,30^ Additionally, the patients in the T + L trial who had ER and/or PR positive tumors by IHC (according to the 2010 ASCO/CAP guidelines)^31^ also received daily oral letrozole (*n* = 10), while ER positive patients in the T-DM1 + P trial did not receive hormone therapy (*n* = 17). The T + P trial excluded ER positive patients. Definitive breast surgery occurred following completion of neoadjuvant therapy and was defined as partial/segmental mastectomy or lumpectomy, total mastectomy, or modified radical mastectomy.

### *HER2/CEP17* FISH Ratio Measurement

The fluorescence in situ hybridization (FISH) assay was performed on paraffinized pre-treatment biopsy tissue samples. HER2+ status was defined as *HER2/CEP17* ratio ≥ 2 by FISH according to ASCO/CAP guidelines.^4^ The *HER2* ratio was calculated by blinded clinical pathologists as the average *HER2/neu* DNA probe signals divided by the average *CEP17* probe signals.

### Pathologic Complete Response Measurement

Surgical pathology specimens collected at the time of definitive breast surgery following neoadjuvant therapy were assessed for pathologic complete response (pCR), defined as the disappearance of all invasive tumor in the breast and axilla (ypT0 ypN0) and axilla.^7-9^ All other patients were defined as non-pCR.

### Statistical Methods

Descriptive statistics on demographic and clinical characteristics were presented as the median with interquartile range (IQR) for continuous variables and frequency with percentages for categorical variables. Between-group differences were assessed using Wilcoxon Rank-Sum test or Pearson’s chi-squared test. The association between *HER2/CEP17* FISH ratio and primary outcome (pCR) was measured using odds ratio (OR) with 95% CIs (CI) by logistic regression modeling. To search for a non-linear relationship between the *HER2* ratio and pCR, a univariable logistic regression model with restricted cubic splines was performed. Based on the non-linear relationship initially observed between the *HER2* ratio and pCR, a second binned analysis categorized the *HER2/CEP17* FISH ratio into 3 groups: Less than 5, 5 to 10.9 and 11 and above. Less than 5 was selected since only 1/13 patients in this group had a pCR, while 11 and above was selected since the nonlinear curve began to slope downward after a HER2 ratio of 11.4. For the *HER2* ratio categorical analysis, associations between *HER2/CEP17* FISH Ratio and pCR status were assessed using logistic regression. Since T-DM1 is an antibody-drug conjugate where emtansine (DM1) has a cytotoxic antineoplastic effect, a subgroup analysis investigating the relationship between the *HER2* ratio and pCR according to treatment regimen was performed using a univariable logistic regression model with restricted cubic splines.

A multivariate analysis was used to control for the most probable confounders, namely treatment regimen. Since patients received different treatment regimens, at times including hormone therapy with letrozole, it was hypothesized this could affect the likelihood of pCR. Due to limited degrees of freedom, no additional covariates were included in the multivariate analysis. Statistical significance was considered at two-sided *P*-value < .05. All statistical analyses were performed using R software version 4.0 in addition to Hmisc and rms software packages.^32^

## Results

### Baseline Characteristics

A total of 56 patients were included in the study (Table 1). All patients were female with a median age at diagnosis of 53 years (IQR 45-63 years). ER status was positive in 52% of patients. The median *HER2/CEP17* FISH ratio was 9.4 (IQR 5.5-13.1). Forty-one percent of patients received ­ado-trastuzumab emtansine/pertuzumab, 11% received trastuzumab/pertuzumab, and 48% received trastuzumab/lapatinib. Forty-nine of the 56 (88%) patients completed the intended course of neo-adjuvant HER2-targeted treatment. Treatments were discontinued in 7 patients due to clinical progression and side effects, including diarrhea, elevated liver enzymes, and renal failure. Of the 7 patients who did not complete the intended treatment course, all continued to surgery. A pCR was achieved in 29 of 56 patients (52%). All patients had histologic grade 2 (*n* = 19) or grade 3 (*n* = 37) tumors, and the rate of pCR between histologic grade was equivalent (52.6% and 51.4%, respectively). Two out of 7 patients who did not complete HER2-targeted neoadjuvant treatment had a pCR.

**Table 1.** Baseline characteristics by pathologic complete response (pCR).

	Pathologic complete response		Total (*N* = 56)	*P-*value
	Yes (*N *= 29)	No (*N *= 27)		
Age, years				.53^a^
* N*	29	27	56	
* *Median	53	52	53	
* *Interquartile range	46-61	44-63	45-63	
Tumor grade, *n* (%)				.93^b^
* *Intermediate	10 (34%)	9 (33%)	19 (34%)	
* *High	19 (66%)	18 (67%)	37 (66%)	
Lymph node involvement, *n* (%)				.66^b^
* *Yes	13 (45%)	15 (56%)	28 (50%)	
* *No	8 (28%)	7 (26%)	15 (27%)	
* *Unknown	8 (28%)	5 (19%)	13 (23%)	
Clinical stage at diagnosis, *n* (%)				.16^b^
* *Stage II	24 (83%)	18 (67%)	42 (75%)	
* *Stage III	5 (17%)	9 (33%)	14 (25%)	
*HER2/CEP17* FISH ratio				.035^a^
* *Median	10.3	5.9	9.4	
* *Interquartile range	8.1-13.1	3.4-12.8	5.5-13.1	
HR status, *n* (%)				.058^b^
* *Positive	12 (41%)	18 (67%)	30 (54%)	
* *Negative	17 (59%)	9 (33%)	26 (46%)	
ER status, *n* (%)				.11^b^
* *Positive	12 (41%)	17 (63%)	29 (52%)	
* *Negative	17 (59%)	10 (37%)	27 (48%)	
PR status, *n* (%)				.30^b^
* *Positive	8 (28%)	11 (41%)	19 (34%)	
* *Negative	21 (72%)	16 (59%)	37 (66%)	
Hormonal treatment if HR positive, *n* (%)				.43^b^
* *Yes	3 (25%)	7 (39%)	10 (33%)	
* *No	9 (75%)	11 (61%)	20 (67%)	
Treatment regimen, *n* (%)				.021^b^
* *T-DM1/Pertuzumab	15 (52%)	8 (30%)	23 (41%)	
* *Pertuzumab/Trastuzumab	5 (17%)	1 (4%)	6 (11%)	
* *Trastuzumab/Lapatinib	9 (31%)	18 (67%)	27 (48%)	
Treatment completed, *n* (%)				.19^b^
* *Yes	27 (93%)	22 (81%)	49 (88%)	
* *No	2 (7%)	5 (19%)	7 (12%)	

^a^Wilcoxon test.

^b^Pearson test.

Abbreviations: *HER2*, human epidermal growth factor receptor 2; CEP17, chromosome 17 centromere; FISH, fluorescence in situ hybridization; TDM1, ado-trastuzumab emtansine.

Patients receiving T + P had a higher median *HER2* ratio (15.5) compared with the other two regimens (*P* = .007) along with the highest rate of pCR (5 out of 6 patients), while T-DM1 + P was the only regimen with an independent positive association between *HER2* ratio and pCR (*P* = .021). T + L had the lowest rate of pCR at 33% consistent with trial data showing lapatinib-containing regimens with lower rates of pCR compared to trials containing T-DM1 or pertuzumab.^16,20,29,33,34^ There was no significant association between pCR and all other baseline characteristics.

### *HER2/CEP17* FISH Ratio and pCR

**Figure 1.**
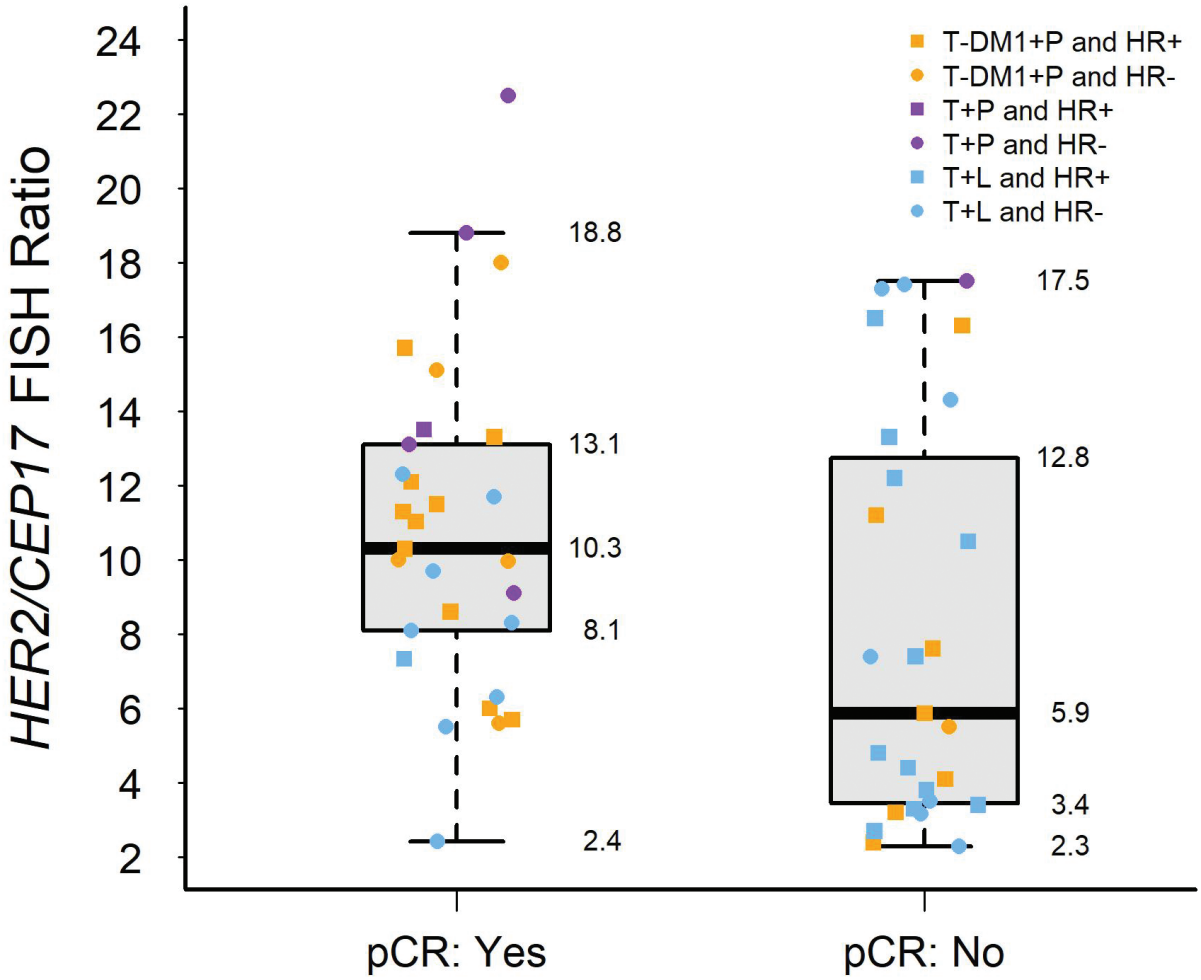
The boxplot of *HER2/CEP17* FISH ratio and pathologic complete response (pCR). Each plotted point represents a patient’s *HER2* ratio at pre-treatment biopsy. The box shape denotes a patient with hormone receptor positive (HR+) breast cancer, while the circle shape represents a patient with HR− breast cancer. The color of each plotted point represents the neoadjuvant treatment regimen the patient received. A pCR is determined from the surgical pathology specimen following neoadjuvant dual anti-HER2 therapy without chemotherapy. Gray box parameters represent the upper and lower quartile *HER2/CEP17* FISH ratio and the line inside the box represents the median *HER2* ratio. The whiskers extend to the furthest points from the upper or lower quartile to 1.5 times the interquartile range or minimum value.

The median *HER2* ratio in patients who achieved pCR was 10.3 (IQR 8.1-13.1), whereas the median *HER2* ratio in patients who did not achieve a pCR was 5.9 (IQR 3.4-12.8, Fig. 1). Only 1 out of 13 patients with a *HER2* ratio less than 5 achieved a pCR.

**Figure 2.**
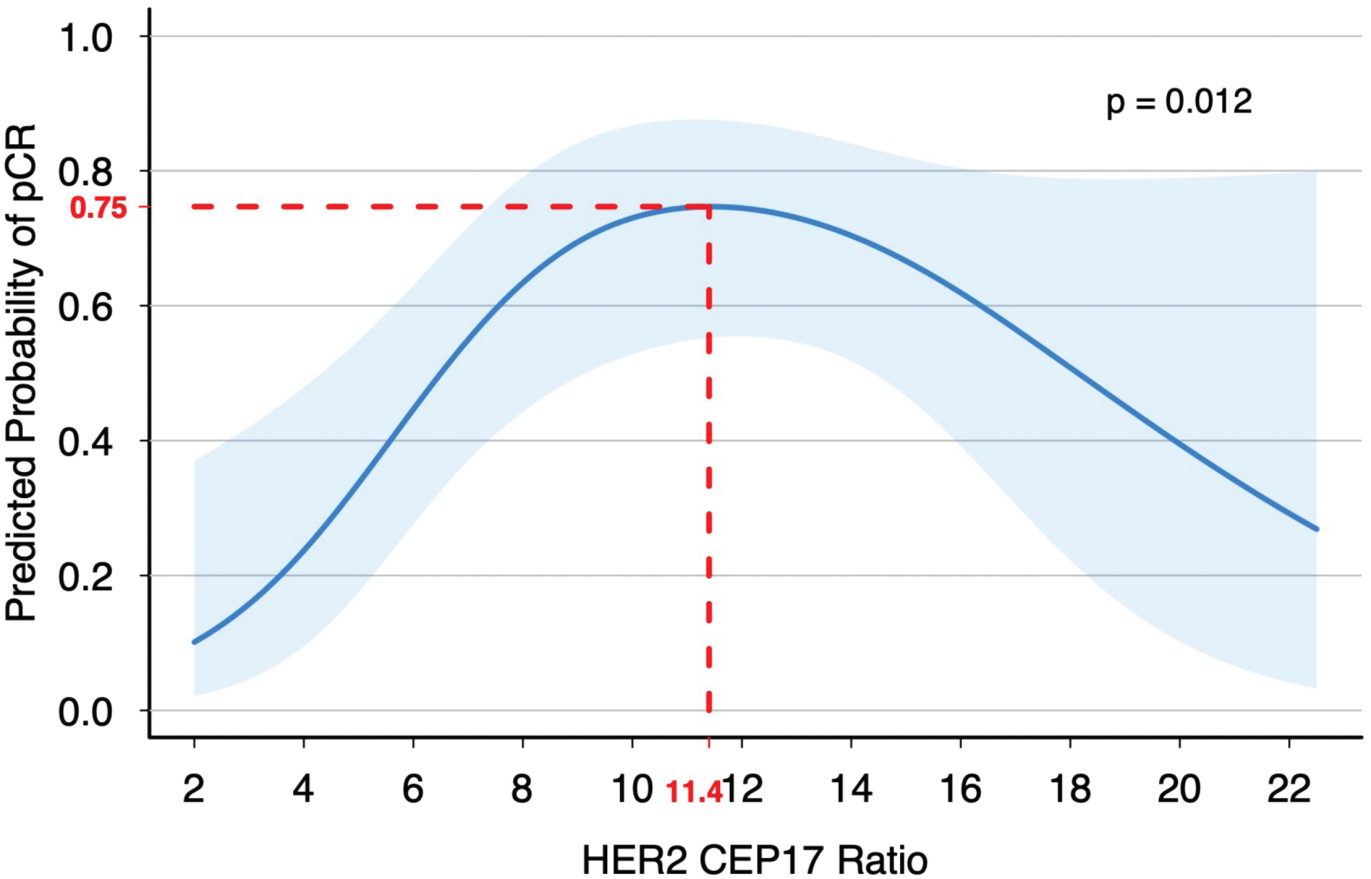
The relationship of the *HER2/CEP17* FISH ratio (continuous variable) to predicted probability of pCR assessed in the logistic regression model.

The median *HER2* ratio was significantly higher in patients who achieved a pCR compared to patients who did not (*P* = .035, Table 1). To examine for both positive and inverse relationships between the *HER2* ratio and pCR, a univariable non-linear logistic regression model with restricted cubic splines was performed. In this model, a *HER2* ratio between 6.5 and 18.1 had a predicted probability of pCR greater than 0.5 (Fig. 2). A *HER2* ratio of approximately 11.4 had the highest predicted probability of pCR, 0.75. The probability of pCR increased until a *HER2* ratio of 11.4 and decreased thereafter. All but two patients receiving T + P or T-DM1+ P with a *HER2* ratio > 11.4 achieved a pCR (10 out of 12 patients). Out of 18 patients with a *HER2* ratio > 11.4, 10 (56%) had a pCR. Of these 10 patients, 5 received T-DM1 + P, 4 received T + P, and 1 received T + L. Eight of 18 patients with a *HER2* ratio > 11.4 did not achieve a pCR, where 6 received T + L, 1 received T + P, and 1 received T-DM1 + P. Overall, 75% of patients with a *HER2* ratio ≥ 12 without a pCR received T + L.

In the logistic regression analysis, patients with a *HER2* ratio in the upper quartile (median *HER2* ratio 13.1) had a 73% predicted probability of achieving a pCR compared to those in the lower quartile (median *HER2* ratio 5.5), who had a 38% probability of achieving a pCR. Patients with a *HER2* ratio in the upper quartile were 4.14 times more likely to have a pCR compared to patients in the lower quartile (95% CI 1.44-11.89; *P* = .012). After controlling for treatment regimen in the multivariate analysis shown in Table 2, the odds of achieving a pCR were 2.87 times higher among patients with an upper quartile *HER2* ratio compared to a lower quartile *HER2* ratio (95% CI 0.9-9.18; *P* = .020).

**Table 2.** The multivariate logistic regression model assessing the odds of achieving a pCR when comparing the median upper and lower quartiles of *HER2/CEP17* FISH ratio after controlling for different treatment regimens.

Covariates	Odds ratio	95% CI
*HER2/CEP17* FISH ratio (13.1 versus 5.5)	2.87	0.9-9.18
Treatment regimen		
* *Trastuzumab + Pertuzumab * *vs. TDM1 + Pertuzumab	6.85	0.27-175.26
* *Trastuzumab + Lapatinib * *vs. TDM1 + Pertuzumab	0.32	0.09-1.14

Abbreviations: pCR, pathologic complete response; *HER2*, human epidermal growth factor receptor 2; CEP17, chromosome 17 centromere; FISH, fluorescence in situ hybridization; TDM1, ado-trastuzumab emtansine.

**Figure 3.**
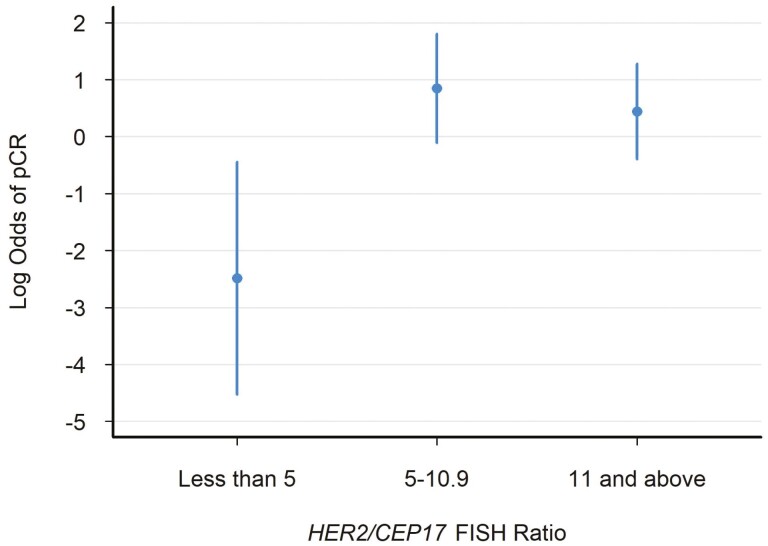
The relationship between the *HER2/CEP17* FISH ratio by categorical group (less than 5, 5 to 10.9, and 11 or greater) and the logarithmic odds of pathologic complete response (pCR) assessed in the logistic regression model. The dot represents the odds ratio and each line represents the 95% CI.

A second logistic regression analysis categorized the *HER2/CEP17* FISH ratio into 3 groups: Less than 5, 5 to 10.9 and 11 and above. Compared to patients with a *HER2* ratio < 5, patients with a *HER2* ratio of 5-10.9 had 28.0 times greater odds of achieving a pCR (95% CI 2.94-266.47; *P *= .004). Patients with a *HER2* ratio  ≥ 11 were still more likely to achieve a pCR compared to patients with a *HER2* ratio < 5 (OR 18.67, 95% CI 2.06-169.3; *P* = .009). Figure 3 depicts these results, where the CIs of *HER2* ratio 5-10.9 and  ≥ 11 mostly overlap.

### *HER2/CEP17* FISH Ratio and pCR According to Treatment Regimen

Since T-DM1 is an antibody-drug conjugate where emtansine (DM1) contains a cytotoxic antineoplastic effect, and T-DM1 + P carried an independent positive association with pCR when comparing median *HER2* ratios, subgroup analyses examining the relationship between the *HER2* ratio and pCR were performed according to treatment regimen received. In patients who received T-DM1 + P (*n* = 23), the rate of pCR was 65% (15/23) where the median *HER2* ratio was 10.0 (IQR 5.8-11.8). The median *HER2* ratio was higher in patients with a pCR compared to patients who did not achieve a pCR (11.0 vs. 5.7; *P* = .029). However, on univariable logistic regression modeling, patients with a *HER2* ratio in the upper quartile (11.8) trended more likely to achieve pCR compared to the lower quartile (5.8) without reaching statistical significance (OR 9.93; 95% CI 1.4-70.4; *P* = .071).

The T + P and T + L groups were combined since only 6 patients received T + P and all 3 drugs were tyrosine kinase inhibitors of HER2. Out of 33 patients who received T + P (*n* = 6) or T + L (*n* = 27), the median *HER2* ratio was 8.3 (IQR 4.4-11.3) and the rate of pCR was 42% (14/33). When comparing patients on dual TKI therapies with pCR and without pCR, respectively, there was no significant difference in the median *HER2* ratio (9.4 vs. 7.4; *P* = .30) or the upper versus lower quartile *HER2* ratio on logistic regression (13.3 vs. 4.4; OR = 3.69; 95% CI 0.78-17.54; *P* = .203).

## Discussion

This is the first study to the authors’ knowledge to analyze the *HER2/CEP17* FISH ratio as a potential biomarker to predict pCR in HER2+ breast cancer patients who received neoadjuvant dual HER2-targeted therapies without cytotoxic chemotherapy. While the 3 trials included in the analysis varied significantly in the median *HER2* ratios and rates of pCR, patients had similar baseline characteristics. Our analysis of the pooled data of the patients enrolled in the 3 studies demonstrates that patients with higher *HER2* ratios may have an increased likelihood of achieving a pCR following dual HER2-targeted therapy without chemotherapy.

While neoadjuvant HER2-targeted therapy with chemotherapy remains the current standard of care for locally advanced HER2+ breast cancer, oncologists may be overtreating a significant proportion of patients who could achieve the same outcomes without chemotherapy. Clinical trials with the de-escalation strategy of chemotherapy omission have been conducted to potentially reduce the financial and pharmacologic toxicities of chemotherapy without endangering patient outcomes. Ubiquitously, trials of doublet HER2-targeted therapy without chemotherapy have achieved a pathologic complete response in 20%-40% of patients.^21^ Though all-comers with HER2+ breast cancer who receive dual anti-HER2 therapy with chemotherapy are more likely to achieve a pCR, the successful identification of patients with HER2 oncogene-dependent tumors who may respond without chemotherapy would lead to a paradigm shift in personalized neoadjuvant treatment for HER2+ breast cancer.

To date, no biomarker has been validated to predict pCR or disease-free survival with chemotherapy-free regimens; however, a number of combinatorial biomarkers have demonstrated that tumors with high levels of *HER2* gene expression are primarily dependent on HER2 signaling.^6,18,22^ The *HER2/CEP17* FISH ratio, assessing the ratio of *ERBB2*:*CEP17* gene amplification via FISH, is already measured from virtually all baseline breast core biopsies where a *HER2* ratio ≥ 2 defines HER2+ breast cancer.^4^ Therefore, if found to be valid biomarker for predicting pCR following neoadjuvant dual HER2-targeted therapy, it may be easily integrated into clinical practice.

The *HER2* ratio has been studied extensively in patients receiving neoadjuvant HER2-targeted therapy with chemotherapy. Retrospective and prospective analyses have shown high *HER2* ratios to be predictive of pCR and ­recurrence-free survival, and they have a tendency to predict overall ­survival.^24-26,35^ One study briefly explored the *HER2* ratio in the neoadjuvant chemotherapy-free setting. In the TBCRC006 trial of neoadjuvant T + L without chemotherapy, Veeraraghavan et al showed that 0 out of 6 patients with a *HER2* ratio < 4 had a pCR, while 29% of 45 patients with a ratio ≥ 4 achieved a pCR.^6^ However, no significant difference in median *HER2* ratio was observed between pCR and no pCR groups. Since 71% of patients with *HER2* ratio ≥ 4 did not have a pCR, it was suggested other mechanisms of treatment resistance were at play. The authors noted that small sample sizes may have explained the failure to detect a significant difference in the rates of pCR based on the *HER2* ratio. It should also be noted that lapatinib is a relatively less effective HER2 targeting drug compared with others, including other small-molecule tyrosine kinase inhibitors, such as neratinib.

To our knowledge, our study is the first which includes patients receiving multiple regimens of neoadjuvant dual HER2-targeted therapy without chemotherapy to explore the *HER2* ratio as a potential biomarker to predict the likelihood of pCR. In our single-center sample of 56 patients, the participants who achieved pCR at the time of surgery following neoadjuvant therapy had a higher baseline median *HER2* ratio (10.3) compared with patients with no pCR (5.9, *P* = .035). The probability of pCR was 73% among patients with a *HER2* ratio of 13.1 compared with a probability of 38% among patients with *HER2* ratio of 5.5 (OR 4.14, 95% CI 1.44-11.89; *P* = .012). When controlling for treatment regimen in the multivariate analysis, this association persisted (OR 2.87; 95% CI 0.9-9.18; *P* = .020).

The probability of pCR was highest with a *HER2* ratio of 11.4 and decreased thereafter. We subsequently performed categorical analysis where *HER2* ratios were grouped according to less than 5, 5-10.9, and 11 or greater. Upon logistic regression, we found that patients with a *HER2* ratio of either 5-10.9 or 11 or greater were more likely to have a pCR than patients with a ratio  < 5. The observed decline in pCR rates with *HER2* ratios  > 11.4 may be explained by different treatment regimen efficacy. Of patients with *HER2* ratios  > 11.4, only 2 out of 8 patients who received T + L had a pCR, while 10 out of 12 combined patients receiving T-DM1 + P or T + P achieved a pCR. Additionally, the T + L regimen had the lowest overall rate of pCR at 33% consistent with trial data showing lapatinib-containing regimens with lower rates of pCR compared with T-DM1 or pertuzumab-based regimens.^16,20,29,33,34^

While a regimen with T-DM1 could be considered to include chemotherapy as it is an antibody-drug conjugate which links trastuzumab to a microtubule inhibitor, it targets HER2 overexpressing cells. As such, it does not lead to significant hair loss and patient-related outcomes data show an improvement in quality of life over traditional cytotoxic chemotherapy.^36,37^ T-DM1 + P was the only single regimen where the *HER2* ratio was associated with pCR, although all patients who received T-DM1 + P tended to have a higher median *HER2* ratio (10.0; IQR 5.8-11.8) compared to patients who received T + L or T + P (8.3; IQR 4.4-11.3), which may confound the association. Nevertheless, the *HER2* ratio remained positively associated with pCR in the multivariate analysis, which adjusted for differences in treatment regimens.

Similar to Veeraraghavan and colleagues’ data, patients with a *HER2* ratio < 4 had a low pCR rate of 10%, while the rate of pCR among patients with a *HER2* ratio > 4 in our study was 60.8%. Though the ASCO/CAP guidelines recommend anti-HER2 therapy for patients with a *HER2* ratio of ≥ 2,^4,5^ data from both studies suggest that the threshold ratio could be set higher for patients with HER2-dependent tumors that may be sensitive to HER2-targeted therapy alone.

Consistent with exploratory analyses from trials of patients receiving anti-HER2 therapy with chemotherapy, our data found a positive association between the *HER2/CEP17* FISH ratio and pCR, where patients with a higher *HER2* ratio have an increased likelihood of achieving pCR following neoadjuvant doublet anti-HER2 therapy without chemotherapy. The exploratory analysis from TBCRC006 showed that patients with both a *HER2* ratio > 4 and intact PI3K pathway were more likely to achieve pCR after receiving neoadjuvant T + L without chemotherapy. Not all of the trials included in our study collected PI3K pathway status on baseline tumor biopsy, limiting our ability to validate the findings from TBCRC006.

Our study has several limitations to be noted. Patient data was gathered from one center; thus, the limited sample size decreased the power of the study. The overall sample size produced wide CIs. The small sample of patients on different regimens may have diminished the ability to robustly control for variations in treatment regimens and treatment durations. Further, the differences surrounding ER status between trials, including inclusion versus exclusion and treatment versus no treatment, may have affected rates of pCR since ER positivity forebodes a lower likelihood of pCR in the neoadjuvant treatment setting.^21^ The data may have been skewed by the 27 patients receiving T + L, since the patients on this trial had lower rates of pCR compared with the other included trials, consistent with published data suggesting that lapatinib has lower response rates in the metastatic setting compared with pertuzumab or T-DM1 and may be a less effective treatment.^16,34^ The T + L trial demonstrated superior rates of pCR among patients who received 24 weeks of neoadjuvant therapy compared to 12 weeks.^20^ Each trial contained a variable duration of neoadjuvant treatment, which may confound the rates of pCR across trials. Our study does not account for the impact of tumor HER2 heterogeneity on pCR rates, since we only investigated the *HER2* ratio from the pre-treatment biopsy sample.^30^

Further studies are warranted to understand whether the *HER2* ratio can be used a predictor of response and achievement of pCR in locally advanced HER2 positive breast cancer. This exploratory study indicates that a higher *HER2* ratio threshold could potentially be used as a cutoff for omitting neoadjuvant chemotherapy and proceeding with dual HER2 directed therapy only. Those patients not achieving a pCR could receive adjuvant chemotherapy and would be eligible to receive trastuzumab emtansine or, if they had received T-DM1 in the neoadjuvant setting, a regimen of chemotherapy with trastuzumab. A more personalized approach such as this could potentially save thousands of patients from the toxic effects of conventional cytotoxic chemotherapy each year.

## Conclusion

In this retrospective single-center study with data from 3 pooled clinical trials, we observed a positive association between the *HER2/CEP17* FISH ratio and pCR among patients with HER2+ breast cancer who received a neoadjuvant anti-HER2 doublet treatment regimen without conventional chemotherapy. Patients who achieved a pCR had a higher median *HER2* ratio compared with patients who did not have a pCR (10.3 vs. 5.9; *P *= .035). Further, the probability of pCR was 73% among patients with a *HER2* ratio of 13.1 compared with a probability of 38% among patients with *HER2* ratio of 5.5 (OR 4.14, 95% CI 1.44-11.89; *P* = .012). The positive association between *HER2* ratio and pCR remained significant after controlling for treatment regimen (*P* = .020). This was the first known study to examine the *HER2* ratio as a predictor of pCR among patients receiving different neoadjuvant anti-HER2 doublet therapies without conventional chemotherapy. Further studies are needed to evaluate *HER2* ratio as a predictor of pCR without cytotoxic chemotherapy in patients with *HER2+* breast cancer.

## Data Availability

The data underlying this article will be shared on reasonable request to the corresponding author.
